# Healthy vaccinee effect by study design

**DOI:** 10.3389/fepid.2026.1837295

**Published:** 2026-09-16

**Authors:** Michael Günther, Harald Walach, Robert Rockenfeller, Matthias Reitzner, Christian Meyer

**Affiliations:** 1 Computational Biophysics and Biorobotics, Institute for Modelling and Simulation of Biomechanical Systems, Universität Stuttgart, Stuttgart, Germany; 2 Friedrich–Schiller–Universität, Jena, Germany; 3 Change Health Science Institute, Basel, Switzerland; 4 Next Society Institute, Kazimieras Simonavicius University, Vilnius, Lithuania; 5 Mathematisches Institut, Universität Koblenz, Koblenz, Germany; 6 Institut für Mathematik, Universität Osnabrück, Osnabrück, Germany; 7 Independent Researcher, Frankfurt/Main, Germany

**Keywords:** all-cause mortality, COVID-19, France, mRNA injection, SARS-CoV-2, vaccination

The study by Semenzato et al. (1) has repeatedly been presented [(2, 3)] as evidence that the French SARS-CoV-2 vaccination campaign since 2021 was a success, quantified by an approximately 25% reduction in all-cause mortality. In this opinion piece, we highlight an issue that, in our view, makes this claim of success untenable.

To this end, we compare the mean annual values of the mortality rates of both 18–59-year-old (hereafter 18–59 years) study groups with the corresponding annual French national all-cause mortality rate. The latter nationwide value was 0.001715 in 2022, calculated from 5-year group data (4, 5), except for the 18–19-year group, for which the size was assumed to be 40% of that of the 15–19-year group. This estimation—derived by summing all group sizes, each weighted by the group’s respective annual all-cause mortality rate ({0.29,0.43,0.48,0.61,0.87,1.29,1.99,3.20,4.91} per thousand), and dividing this weighted sum by the sum of the group sizes (35,296,713)—closely matches the figure given in an online comment to Semenzato et al. (1) (by X. Azalbert, C. Perronne, M. Zizi, 11 December 2025).

Semenzato et al. (1) reported the following group-mean annual rates, averaged across 39 months: 0.001694 for the unvaccinated (*unvacc*) and 0.001330 for the vaccinated (*vacc*). These annual rates were calculated by dividing the sum of all deaths counted in either group during 39 months (1, eTable 2; eFigure 5)—32,662 (*unvacc*) and 98,429 (*vacc*)—by the respective group sizes (1, eTable 4) (see also Table 1)—5,932,443 and 22,767,546—and scaling by 3.25 $\left(\frac{39\;\text{months}}{12\;\text{months}}\right)$. The mortality rate of the entire (18–59 years) *unvacc* group in (1) being slightly lower than that of the French population may be explained by the members of the group being younger on average (Table 1), a factor that applies to the *vacc* group to a lesser degree.

**Table 1:** Sizes of age groups within the overall age group (18–59 years), and percentage fractions therein, at the start of follow-up on 1 November 2021, as in (1).

Age group (year)	French population (4), n (%)	*unvacc* (1, eTable 4), n (%)	*vacc* (1, eTable 4), n (%)
18–29	9,381,338 (26.6)	1,777,782 (30.0)	6,553,907 (28.8)
30–39	8,370,024 (23.7)	1,737,069 (29.3)	5,535,008 (24.3)
40–49	8,620,681 (24.4)	1,322,270 (22.3)	5,889,066 (25.9)
50–59	8,924,670 (25.3)	1,095,322 (18.4)	4,789,565 (21.0)
Sum	35,296,713	5,932,443	22,767,546

The study’s (1) overall number of patients analyzed (reimbursed in 2020 by the French National Healthcare Data System, Système National des Données de Santé, SNDS) was approximately 33.9 million (1, eFigure 2), while the total French population in this age group was 35 million in 2020 and 35.3 million in 2022. We conclude that the patient population analyzed in the study closely represents the French population. Approximately 4.05 million 18–59-year patients were excluded from the study because they were vaccinated before 1 May 2021. When combined with two smaller excluded groups—those vaccinated later (about 0.64 million) and those who received non-mRNA-based vaccines (about 0.47 million), respectively (see further below; total *vaccX* group)—these individuals were on average older or more vulnerable (Table 1). Given the French nationwide all-cause mortality rate (0.001715), the mortality rate of the *unvacc* group (0.001694) closely matches this nationwide average, while the *vacc* group exhibits a substantially lower rate (0.001330). Consequently, the *vaccX* group of size 5,162,545 (1, eFigure 2)—comprising those vaccinated early, those vaccinated during the 6-month lag period (1 May to 31 October 2021), and recepients of non-mRNA vaccines—must have exhibited a mortality rate substantially exceeding the nationwide average.

An all-cause mortality rate, X, of the latter *vaccX* group can be derived to be approximately twice as high as the nationwide value, namely, X=0.003437, which is not surprising considering that the *vaccX* group must be older (and likely more vulnerable) on average than both the *unvacc* and *vacc* groups (see Table 1). This X value follows from requiring the sum of annual counts (here, using consistent 2022 data) of all-cause deaths within each of the *three* study groups, the *unvacc*, *vacc*, and *vaccX* groups, to equal the annual count of deaths expected from the nationwide all-cause rate for all three groups combined, thereby assuming that the latter group of all patients actually analyzed in (1) (comprising explicitly the *unvacc*, *vacc* and implicitly the *vaccX* groups: 5,932,443+22,767,546+5,162,545=33,862,534) is closely representative of all individuals aged 18–59 years in France. That is, with the *vacc* and *unvacc* rates of 0.001330 and 0.001694, respectively, we found

X=(0.001715⋅33,862,534−0.001694⋅5,932,443−0.001330⋅22,767,546)/5,162,545=0.003437

for the *vaccX* group. The mortality rate V of the combined *vacc&vaccX* group of all vaccinated would be

V=(0.001715⋅33,862,534−0.001694⋅5,932,443)/(22,767,546+5,162,545)=0.001719.

One may object that an additional 641,910 patients (1, eFigure 2) “left” the *unvacc* group during the 39 months of follow-up due to being vaccinated after 31 October 2021, meaning their group size should rather be close to 5,290,533=5,932,443−641,910 than 5,932,443. In fact, most of these “leavers” were vaccinated during the first 3 months of follow-up, with 5,400,061 *unvacc* remaining (1, eFigure 5), meaning 532,382=5,932,443−5,400,061 actually “left” (or died). The study’s *unvacc* mortality rate would then be estimated by ignoring the first 3 months of follow-up, which yields $\frac{32,662-2,990}{5,400,061}\cdot \frac{12\;\text{months}}{36\;\text{months}}=0.001832$ instead of 0.001694; the group size of the *vaccX* cohort would then be 5,694,927=5,162,545+532,382 instead of 5,162,545, and the correspondingly corrected value of the *vaccX* rate would be

$$X_{\mathrm{cor}}=(0.001715\cdot 33,862,534-0.001832\cdot 5,400,061-0.001330\cdot 22,767,546)/5,694,927=0.003143$$

instead of X=0.003437, which is still by a factor of 1.83 higher than the French nationwide all-cause mortality rate of 0.001715. The correspondingly corrected *vacc&vaccX* rate would be

$$V_{\mathrm{cor}}=(0.001715\cdot 33,862,534-0.001832\cdot 5,400,061)/(22,767,546+5,694,927)=0.001693$$

instead of V=0.001719.

The corrected mortality rate of 0.001832 in the *unvacc* group after the 3-month follow-up delay being higher than 0.001694 may reflect disproportionate vaccination among younger individuals during those 3 months, resulting in their removal from the *unvacc* group, and a corresponding increase in the average mortality rate of the remaining cohort. The study’s reported data (1) lack detailed information about age profile shifts during follow-up in the *unvacc* group. At least during the summer of 2021, during the 6-month lag period, an increasing accumulation of younger individuals in the *vacc* group was indeed observable (1, eTable 4).

The study’s (1) *vacc* rate of 0.001330, which is lower than the *unvacc* rate ranging between 0.001694 and 0.001832, would yield a hazard ratio (HR) between 0.785 and 0.725. Therefore, the homogeneously positive HR effect of vaccination asserted by Semenzato et al. (1) is fully explainable by the exclusion criteria and constitutes a healthy vaccinee effect of a special kind, primarily driven by the disproportionate exclusion of the oldest (Table 1) and more vulnerable individuals (6, Figure 3a: “… et personnes vulnérables”), (1, author response by M. Zureik et al., 19 December) from the analyzed intervention group (*vacc*). The complete population of (nearly) *all* vaccinated French individuals aged 18–59 years comprises both the cohort explicitly analyzed by Semenzato et al. (1) from 1 May 2021 onward (*vacc*) *and* the excluded vaccinated individuals (vaccX), those vaccinated between 1 May and 31 October 2021, and those who received a non-mRNA vaccine. Once the all-cause mortality counts of this complete vaccinated population are factored in, the conclusion that SARS-CoV-2 vaccination is associated with, or induces, a reduction in all-cause mortality is no longer tenable.
